# The gut microbiota in mouse models of inflammatory bowel disease

**DOI:** 10.3389/fcimb.2014.00028

**Published:** 2014-02-28

**Authors:** Kalliopi K. Gkouskou, Chrysoula Deligianni, Christos Tsatsanis, Aristides G. Eliopoulos

**Affiliations:** ^1^Molecular and Cellular Biology Laboratory, Division of Basic Sciences, University of Crete Medical SchoolHeraklion, Greece; ^2^Laboratory of Translational Medicine and Experimental Therapeutics, University of Crete Medical SchoolHeraklion, Greece; ^3^Department of Clinical Chemistry, University of Crete Medical SchoolHeraklion, Greece; ^4^Laboratory of Cancer Biology, Institute of Molecular Biology and Biotechnology–FORTHHeraklion, Greece

**Keywords:** microbiota, colitis, mouse models, IBD, Crohn's disease

## Abstract

The intestine and the intestinal immune system have evolved through a symbiotic homeostasis under which a highly diverse microbial flora is maintained in the gastrointestinal tract while pathogenic bacteria are recognized and eliminated. Disruption of the balance between the immune system and the gut microbiota results in the development of multiple pathologies in humans. Inflammatory bowel diseases (IBD) have been associated with alterations in the composition of intestinal flora but whether these changes are causal or result of inflammation is still under dispute. Various chemical and genetic models of IBD have been developed and utilized to elucidate the complex relationship between intestinal epithelium, immune system and the gut microbiota. In this review we describe some of the most commonly used mouse models of colitis and Crohn's disease (CD) and summarize the current knowledge of how changes in microbiota composition may affect intestinal disease pathogenesis. The pursuit of gut-microbiota interactions will no doubt continue to provide invaluable insight into the complex biology of IBD.

## Introduction

The lower gastrointestinal tract of healthy adult humans contains more than 100 trillion bacteria (Ley et al., 2008), termed the gut “microbiota,” which are involved in complex interactions with host mucosal epithelial and immune cells and shape fundamental physiological processes such as digestion, energy homeostasis, and development of gut-associated lymphoid tissues (Bakhtiar et al., 2013). Surface antigens and metabolic end-products of gut microbiota modulate the activation of resident immune cells and the production of cytokines which protect against potential pathogens (Cario, 2013). However, this homeostatic relationship is perturbed in inflammatory bowel diseases (IBD), a group of chronic relapsing and remitting disorders of the gastrointestinal tract manifesting as Crohn's disease (CD) or ulcerative colitis (UC). UC usually affects only the rectum and shows continuous inflammation, whereas CD may occur anywhere along the gastrointestinal tract and is characterized by discontinuous lesions in the intestinal wall.

One of the most important and devastating complications of the long-standing inflammation in IBD is colorectal cancer development. The first case of UC-associated carcinoma of the intestine was reported by Crohn and Rosenberg (1925), and CD was connected to cancer in 1945 (Warren and Sommers, 1948). Subsequent studies confirmed that patients with IBD, especially UC, have increased risk for developing colorectal cancer and this risk increases further with the severity of inflammation (reviewed in Danese and Mantovani, 2010; Rubin et al., 2012).

The realization of the intimate relationship between the microbiota and intestinal homeostasis has spurred large collaborative efforts aiming to identify and characterize the microorganisms which associate with health and disease in humans. The European MetaHIT [Metagenomics of the Human Intestinal Tract, (Qin et al., 2010)] project and the Human Microbiome Project [HMP, (Peterson et al., 2009)] explore multi-“omic” data to define the role of human microbiome in health and disease along with the development of a reference set of microbial genome sequences. However, experimental model systems such as the mouse and Drosophila continue to provide critical insight into how host-microbiota homeostasis is established, maintained or perturbed (Kostic et al., 2013).

**Figure 1:**
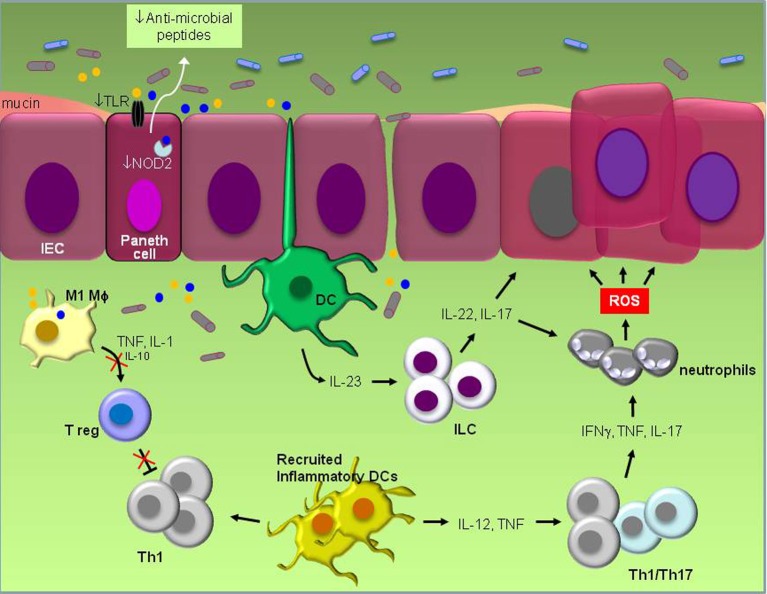
**Schematic representation of known pathogenic events in experimental IBD.** Defective TLR and NOD signaling in Paneth epithelial cells leads to reduced “sensing” of bacterial products (yellow and blue circles) and reduced production of anti-microbial peptides. The ensuing disruption of microbiota balance which may also be influenced by the frequent use of antibiotics and/or diet stimulates inflammation that is largely orchestrated by resident dendritic cells (DCs). Their activation by products of pathogenic bacteria induces IL-23 which in turn engages innate lymphoid cells (ILC) to produce IL-22 and IL-17. Inflammation also results in the recruitment of inflammatory DCs which secrete IL-12 and TNF and increase IFNγ, TNF and IL-17-producing Th1/Th17 cells. Cytokines secreted by ILCs and Th1/Th17 cells promote both the recruitment of neutrophils that produce DNA-damaging reactive oxygen species (ROS) and the survival of intestinal epithelial cells (IEC) by the engagement of STAT3 signal transduction, eventually leading to malignant transformation. Suppression of regulatory T cell (T_reg_) activity by pro-inflammatory M1 macrophages which secrete high TNF and IL-1 but low IL-10 levels unleashes inflammation and allows macrophages to produce oxidative products and mutagens which are believed to contribute to carcinogenesis. Reduced production of mucus by Goblet cells impacts on microbial composition and gastrointestinal barrier function.

Herein, we review the phenotypic, cellular, and molecular characteristics of some of the most widely-used mouse models of experimental IBD and colitis-associated cancer (CAC) and the impact of microbiota on these pathologies (Figure 1).

## Chemical and genetic mouse models of inflammatory bowel disease and colitis-associated colon cancer

### Dextran sodium sulfate-induced colitis

An established model of IBD employs the chemical Dextran Sodium Sulfate (DSS). DSS administered to the drinking water in repeated cycles triggers a state of chronic intestinal inflammation by binding to medium-chain-length fatty acids present in the mouse colon, inducing disruption of colonic epithelial barrier (Laroui et al., 2012). The ensuing tissue damage allows exposure of innate immune cells to commensal bacteria accompanied by a robust Th1-type immune response to eliminate infiltrating pathogens and promote tissue healing. Multiple cell types participate in the pathogenesis of DSS-induced colitis including gut epithelial cells, CD4^+^ and CD8^+^ T lymphocytes, regulatory T cells, neutrophils and macrophages, resembling the pathogenic events in human colitis. Mucosal macrophages may prime the local inflammatory response through both phagocytosis of DSS and activation by bacteria products. The contribution of macrophage polarization phenotype to the development of CAC has been described using this model including the demonstration that Akt2 deficient mice are partly protected from DSS-induced colitis because of a macrophage phenotype shift from M1 to M2 in the colonic mucosa (Arranz et al., 2012).

Chronic inflammation induced by prolonged administration of DSS results in malignancy only in a small proportion of animals (Okayasu et al., 1990, 1996) but adenocarcinoma development readily occurs upon intraperitoneal injection of the mutagen azoxymethane (AOM) followed by repeated DSS cycles (reviewed in Wirtz et al., 2007; Chen and Huang, 2009). AOM is metabolized *in vivo* to methylazoxymethanol (MAM) by cytochrome P450 (Sohn et al., 2001). MAM and its derivatives are direct DNA mutagens although tumor formation requires additional cellular and molecular events associated with chronic inflammatory imbalance. Indeed, the degree of inflammation correlates with the development of dysplasia in minor lesion aberrant crypt foci and is linked to the nuclear translocation of β-catenin (Cooper et al., 2000). Impairment of indoleamine 2,3 dioxygenase-1 (IDO-1) activity, a molecule which catabolizes tryptophan in the kynurenine pathway and is expressed in inflamed and neoplastic intestinal epithelial cells, reduces nuclear β-catenin and cell proliferation (Thaker et al., 2013). Inflammatory cytokines such as TNF, IL-6, and IL-1α which have been implicated in human IBD and IBD-associated colorectal carcinogenesis, also largely dictate the outcome of AOM/DSS-induced pathology (Becker et al., 2004; Van Hauwermeiren et al., 2013; Bersudsky et al., in press). Interestingly, mice deficient in myeloid translocation gene related-1 (MTGR1) are resistant to AOM/DSS-induced CAC despite the preservation of an active inflammatory infiltrate. Tumor resistance in these animals arises from increased malignant cell death and impaired wound-healing (Barrett et al., 2011), suggesting that in addition to the severity of inflammation, AOM/DSS-induced carcinogenesis depends on apoptosis and wound-healing regulatory pathways.

Mutations in p53 are abundant in both sporadic and IBD-associated colorectal cancer in humans, suggesting a pivotal role for this tumor suppressor in intestinal disease pathogenesis. However, whereas p53 mutations are late genetic events in sporadic CRC, they are observed in inflamed colonic tissue well before neoplastic lesions become detectable (Hussain et al., 2000). Thus, p53 mutations probably have an initiating role in human IBD-associated cancer. In the mouse colon, AOM/DSS-induced pathology is largely amplified by either mutations or loss of WT p53. Knock-in mice carrying a germline mutated p53 allele encoding p53R172H, the mouse equivalent of the human hot spot mutant p53R175H (Lang et al., 2004), develop adenocarcinomas even in the absence of AOM treatment (Cooks et al., 2013). The accelerated tumorigenesis in these animals results from a combination of amplified and prolonged inflammation and augmented capacity of mutated p53-containing epithelial cells to evade apoptosis. P53-deficient or p53^+/−^ mice also develop multiple tumors upon exposure to DSS without the requirement of AOM administration (Fujii et al., 2004; Chang et al., 2007). Therefore, AOM/DSS induces a state of chronic intestinal inflammation which progresses to cancer with molecular, histopathological and phenotypic characteristics that resemble the human disease.

Another carcinogen used in combination with DSS is 1, 2-dimethylhydrazine (DMH). DMH is metabolized in liver and its derivatives induce the production of diazonium by gut epithelial cells. The aforementioned metabolite exerts mutagenic effects through oxidative stress and methylation events (Hamiza et al., 2012).

### TNBS-induced inflammatory bowel disease

Intrarectal administration of the contact sensitizing allergen 2,4,6-trinitrobenzenesulfonic acid (TNBS) initiates acute T cell-mediated, IL-12 driven intestinal inflammation (Scheiffele and Fuss, 2002; Neurath and Finotto, 2009). Ethanol is required to disrupt the mucosal barrier, whereas TNBS is proposed to haptenize microbiota or colonic autologous proteins rendering them immunogenic. The overall phenotypic and histopathological features of TNBS-induced colitis mostly resemble those characterizing CD. Recently, the TNBS model was used for the identification of rVEGF164_b_, a VEGF-A isoform, as an inhibitory molecule of angiogenesis in IBD (Cromer et al., 2013). Thus, TNBS is considered as a suitable model to study both gut inflammation and the mechanism involved in colonic healing in IBD. Using this model we have recently described the efficacy of antisense oligonucleotides targeting CD40, a TNF family receptor that triggers Th1 and innate immune responses upon stimulation by its ligand, in treating early stage and established colitis (Arranz et al., 2013).

### Adenomatous polyposis coli mutation-induced adenoma model

Mutations in the Adenomatous polyposis coli (APC) gene in humans are critically involved in familial adenomatous polyposis (FAP) and represent an early genetic aberration in sporadic colorectal cancer (Liang et al., 2013). The multiple intestinal neoplasia (Min) mouse, one of the first genetic models used to study intestinal cancer in rodents, bears a point mutation in the Apc gene (Apc^min/+^) and develops numerous adenomas. Exposure of Apc^min/+^ mice to DSS alone mimics CAC and results in accelerated tumorigenesis (Tanaka et al., 2006). In addition to inflammation, Apc^min/+^-induced carcinogenesis can be influenced by oxidative stress. Thus, Cheung et al. (2012) showed that ablation of nuclear factor-erythroid 2 related factor 2 (Nrf2) attenuates anti-oxidative stress pathways and increases proliferation in the intestinal crypts leading to enhanced intestinal carcinogenesis in Apc^min/+^ mice. This observation is pertinent to the role of gut microbiome in disease pathogenesis, identifying microbial metabolites as modulators of carcinogenesis in part through induction of chronic oxidative stress (Arthur et al., 2012).

### IKK-γ (NEMO) deficiency in intestinal epithelial cells

Intestinal epithelial-cell-specific inhibition of NF-κB through conditional ablation of NEMO/IKKγ, the regulatory subunit of the IKK signaling complex essential for NF-κB activation, spontaneously causes severe chronic intestinal inflammation in mice (Nenci et al., 2007). Histological examination of colon sections from these animals revealed extensive apoptosis of colonic epithelial cells leading to disruption of epithelial integrity and translocation of bacteria from the lumen into the mucosa. Notably, these mice exhibit reduced expression of defensin-3, an antimicrobial peptide primarily produced by specialized intestinal epithelial cells, called Paneth. Low defensin copy number has been reported to correlate with predisposition to IBD in humans (Wehkamp et al., 2006) and unpublished data from our laboratory suggest that defensin expression is higher in the proximal compared to distal colon reflecting their differential susceptibility to DSS-induced pathology (Gkouskou and Eliopoulos, in preparation).

### Interleukin-10 (IL-10)-dependent inflammatory bowel disease

Genome-wide association studies have identified SNPs flanking the IL-10 gene to be linked to UC (Franke et al., 2008). IL-10-deficient mice exhibit intolerance to their intestinal microbiota, have altered responses to inflammatory or autoimmune stimuli and develop spontaneous enterocolitis and adenocarcinoma (Sturlan et al., 2001). A similar intestinal phenotype was observed in mice with a T cell specific IL-10 deficiency, underscoring the importance of T cell derived IL-10 and IL-10-dependent regulatory T-cells in the regulation of mucosal T cell responses and disease pathogenesis (Erdman et al., 2003).

### T cell adoptive transfer model

Initially developed by the group of Fiona Powrie (Powrie et al., 1994), mouse CD4^+^ T lymphocytes which express high CD45RB (CD4^+^CD45RB^Hi^) can be adoptively transferred into immunodeficient SCID or RAG1/2^−/−^ mice, where they traffic to the intestine and induce gut inflammation. Recipient mice repopulated with CD4^+^CD45RB^Lo^ T cells or total CD4^+^ T lymphocytes do not develop colitis, despite their ability to colonize the host gut. This phenomenon is attributed to the presence of CD25^+^FoxP3^+^ regulatory T cells within the CD4^+^CD45RB^Lo^ population (Read et al., 2000) and adoptive transfer of CD4^+^CD25^−^ T cells has thus been proposed as the most suitable T cell transfer model of enterocolitis (Kjellev et al., 2006). IL-10 appears to have an important role in the pathogenesis of the disease in this model as SCID mice administered both CD4^+^CD45RB^Hi^ and regulatory T cells together with anti-IL-10 receptor antibodies develop colitis (Kjellev et al., 2006).

## The gut microbiota in mouse models of IBD

Several lines of evidence support a role for the microbiota in experimental colitis. Early studies reported a significant increase in members of *Bacteroidaceae* and *Clostridium* spp. families, in particular *Bacteroides distasonis* and *Clostridium ramosum*, in the intestines of mice exposed to DSS (Okayasu et al., 1990) (Table 1). Subsequent reports showed elevated 16S rRNA gene copy numbers of the mucin-degrading Gram-negative bacterium *Akkermansia muciniphila* and of *Enterobacteriaceae* to correlate with disease activity in mice administered DSS (Hakansson et al., 2014). A breakthrough in appreciating the major impact of gut microbiota on disease pathogenesis came by the observations that treatment with antibiotics or germ-free breeding of various mouse models of IBD is associated with significantly less severe bowel inflammation and carcinogenesis. Thus, Dove and colleagues showed that *Apc*^Min/+^ mice housed in germ-free environment display more than 50% reduction in tumor development compared to the same animals housed in standard specific pathogen-free (SPF) conditions (Dove et al., 1997). IL-10 deficient mice were also found to be resistant to spontaneous colitis when kept in germ-free environment (Sellon et al., 1998).

**Table 1:** **Microorganisms reported to associate with IBD in the mouse**.

**Type of disease or model**	**Microorganisms**	**Final effect**	**References**
DSS colitis	*Bacteroides distasonis, Clostridium ramosum, Akkermansia muciniphila, Enterobacteriaceae*	Increased numbers correlate with acute and chronic ulcerative colitis	Okayasu et al., 1990; Hakansson et al., 2014
Colitis in IL-10 deficient mice	*Enterobacteriaceae* and adherent-invasive *E. coli*	Increased numbers correlate with inflammation (*Enterobacteriaceae)* and cancer *(E. coli)*	Arthur et al., 2012; Yang et al., 2013b
Colitis in ApcΔ^468^/IL-10^−/−^ mice	*Bacteroides* and *Porphyromonas* genera	Increased numbers correlate with inflammation and colon polyposis	Dennis et al., 2013
TNBS colitis	*Enterobacteriaceae, Bacteroides*	Increased numbers correlate with inflammation	Ettreiki et al., 2012

*Differences in intestinal microbiota composition due to different housing conditions have been reported (Yang et al., 2013b)*.

Analysis of different classes of antibiotics indicated differential and localized roles of bacteria species in the establishment and perpetuation of colitis in IL-10^−/−^ mice after colonization with SPF bacteria. Ciprofloxacin was found to be most effective in caecal inflammation by controlling aerobic organisms, including *E. coli* and *E. faecalis*, whereas metronidazole was preferentially active in the colon and selectively decreased anaerobic bacteria and *Bacteroides* spp. (Hoentjen et al., 2003). Interestingly, whereas induction of colitis in IL-10^−/−^ mice born under SPF conditions and in mice exposed to DSS is prevented by ciprofloxacin and metronidazole respectively, these antibiotics have minimal effect after the onset of colitis (Hans et al., 2000; Madsen et al., 2000). In contrast, vancomycin-imipenem reduces total luminal bacteria, eliminates specific aerobic and anaerobic organisms and effectively treats established disease (Hoentjen et al., 2003). These results suggest that some intestinal bacteria species may orchestrate the initiation of inflammation whereas other subsets may have a role in perpetuating colitis (Rath et al., 2001). In line with this notion, colonic polyps developed in ApcΔ^468^/IL-10^−/−^ mice are significantly enriched in two genera of the phylum *Bacteroidetes*, namely *Bacteroides* and *Porphyromonas* as compared with uninvolved tissue (Dennis et al., 2013) (Table 1). The interplay between oncogenes and microbiota species in the development of gut pathologies is also highlighted by studies in Drosophila which have demonstrated that the human pathogen *Pseudomonas aeruginosa* synergizes with the RasV12 oncogene to induce intestinal dysplasia and metastasis-like phenotype (Apidianakis et al., 2009; Bangi et al., 2012).

Further evidence supporting the significance of microbes in colitis development has been provided by studies describing a communicable form of colitis induced by deficiency of T-bet in cells of the innate immune system. T-bet is a transcription factor with a pivotal role in the development of Th1 cells and in the regulation of adaptive and innate immune responses. Loss of T-bet in mice lacking B and T cells (*T-bet*^−/−^/*RAG-1*^−/−^) results in spontaneous colitis which is transmissible to wild-type animals (which express T-bet) upon cross-fostering or co-housing (Garrett et al., 2007).

Nutrition plays an important role in the establishment of microbial flora which in turn affects metabolism of several macro- and micronutrients. For example, a high *Firmicutes* to *Bacteroidetes* ratio and low microbial diversity is indicative of a high-calorie diet and obesity in humans (Ley et al., 2006). A telling example of how genetics, microbiota and the immune system may interact to promote chronic gut inflammation is highlighted by a recent study by Devkota et al. (2012) which demonstrated that the ingestion of saturated fat by IL-10^−/−^ mice induces a more severe form of chronic colitis compared to the disease that normally develops in these animals. This diet was shown to stimulate the formation of taurocholine-conjugated bile acids leading to intestinal dysbiosis characterized by the overgrowth of the rare sulfate-reducing pathogenic bacteria *Bilophilia wadsworthia* (Devkota et al., 2012). The modulation of microbiota species and density has also highlighted the important role of bacteria in gut homeostasis and disease. Thus, administration of VSL#3 probiotics, a mixture of *Lactobacillus, Bifidobacterium* and *Streptococcus salivarious* strains, has shown to confer beneficial effects on various mouse models of colitis and in humans suffering from IBD (Isaacs and Herfarth, 2008). Intriguingly, VSL#3 does not reduce colitis-associated colon cancer in the mouse (Arthur et al., 2013).

Direct evidence for the role of pathogenic bacteria in IBD has been provided by studies using the aerobic bacterium *Helicobacter hepaticus*. Immunodeficient RAG^−/−^ mice infected with *H. hepaticus* and treated with AOM develop invasive colon carcinoma after 3–5 months, at the sites of highest inflammation in the colon and cecum (Fox et al., 2011). This model has also assisted in the identification of a genetic interval on the telomeric part of mouse chromosome 3 designated *Hiccs* (*Helicobacter hepaticus*-induced colitis and associated cancer susceptibility), which harbors 8 genes and 5 micro RNAs and confers protection against *H. hepaticus*-induced chronic colitis and inflammation-driven colon cancer (Boulard et al., 2012).

What are the mechanisms by which bacteria dysbiosis triggers inflammatory bowel disease? Several studies have highlighted a prominent role for TLR and NOD family members as key sensors of and responders to microbe-associated molecular patterns. The effects of *Nod2* mutations are of particular interest because they have been implicated in human IBD and *Nod2* knockout mice have diminished ability to prevent intestinal colonization of pathogenic bacteria (Petnicki-Ocwieja et al., 2009; Couturier-Maillard et al., 2013). Impaired TLR and NOD function in Paneth epithelial cells affects their capacity to produce antimicrobial factors which kill pathogenic bacteria, resulting in a shift in the composition of gut microbiota (Figure 1). Frequent use of antibiotics or personal habits, including diet may also influence this shift. The concomitant release of ATP, other metabolic products and DNA by microbia (Atarashi et al., 2008; Hall et al., 2008) may lead to increased production of IL-23 by resident monocytes, such as dendritic cells, which in turn stimulates innate lymphoid cells to secrete IL-17, IL-22, and IFNγ (Buonocore et al., 2010). IL-17 is of particular relevance to colitis as it is linked to reduced regulatory T cell (T_reg_) activity. Resident T_reg_ produce IL-10 which inhibits Th1 cells and monocyte effector functions associated with inflammation. Suppression of T_reg_ activity thereby unleashes inflammation, leading to a switch in the differentiation program of Ly6C^hi^ monocytes from anti-inflammatory M2 macrophages to inflammatory dendritic cells and M1 macrophages in the colon (Rivollier et al., 2012) which produce a plethora of pro-inflammatory cytokines, oxidative products and mutagens such as *trans*-4-hydroxy-2-nonenal (4-HNE) (Yang et al., 2013a). Reactive oxygen species (ROS) generated by recruited neutrophils may also cause DNA damage in epithelial cells.

The production by pathogenic bacteria of secondary bile acids that have carcinogenic effects is believed to contribute to the dysbiosis-inflammation-tumorigenesis axis (Sommer and Backhed, 2013). Additional host genetic factors may influence the cross-talk between microbiota and IBD. For example, production of mucus by Goblet cells, especially mucin 2 (MUC2), has a significant impact on microbial composition and gastrointestinal barrier function. Altered MUC2 expression and/or glycosylation leads to accompanying intestinal pathologies, including IBD and colon cancer (Yang et al., 2008).

## Conclusions and future directions

In the intestine, the symbiotic relationship between the host and the microbiota influences nutrition, metabolism, immune system functions, development and normal physiology, as well as susceptibility to IBD and CAC. Accumulating experimental, epidemiological, and clinical evidence highlights the potential of targeting the dysbiosis-inflammation-tumorigenesis axis for the development of new therapeutic strategies for IBD and colorectal cancer. Much of the current knowledge of the regulation of this axis comes from studies exploring the effects of particular pathogenic bacteria using chemical or genetic models of CAC. High-throughput human microbiome studies confirm that the genetic make-up, environmental factors and personal habits influence the bacteria communities among individuals; however, further studies are warranted to fully appreciate how a particular microbiota is established and orchestrates the immune responses toward the development of colitis and CAC. The establishment of “humanized” gnotobiotic mice, animals that carry only human-derived gut microbes (Turnbaugh et al., 2009) is expected to improve human disease modeling and provide further insight into how environmental factors, including diet, may influence the microbiota and shape gut physiology and disease pathogenesis. Similarly, it would be important to assess changes in the gut flora during aging and evaluate their impact on IBD susceptibility. In line with this notion, recent studies in Drosophila show that immunosenescence associated with aging results in dysbiosis and triggers an inflammatory response which promotes intestinal stem cell over-proliferation and dysplasia (Guo et al., 2014). Further studies are also needed to determine whether changes in particular microbiota species induced by inflammation may impact on progression to cancer.

Future research could also lead to the development of beneficial (probiotic) microbes or inhibitors of specific microbes and/or their products which “normalize” the intestinal flora and can improve human health. As the current repertoire of probiotics is limited, further studies to explore the potential of fecal microbiota transplantation (FMT) therapy, the infusion of fecal bacteria from a healthy individual into a recipient patient, for the treatment of intestinal disorders are warranted. FMT has demonstrated tremendous efficacy in treating refractory *Clostridium difficile* infection, and there are case reports of successful management of UC using FMT in humans (Lemon et al., 2012). A more focused approach requires the identification of species or bacterial products and metabolites which normalize the inflamed gut mucosa. In this regard, the isolation of 17 human clostridia species and the discovery of microbial-derived short-chain fatty acids that can stimulate the expansion of T_reg_ cells in mice (Atarashi et al., 2013; Smith et al., 2013) opens up new therapeutic options for the treatment of IBD.

The microbiome plays an important role in immunity and energy metabolism and will thus be important to determine if the microbial gut ecology may also impact on non-gastrointestinal diseases, including obesity, cancer and neurological disorders.

### Conflict of interest statement

The authors declare that the research was conducted in the absence of any commercial or financial relationships that could be construed as a potential conflict of interest.
